# Epidermal transient receptor potential vanilloid 1 in idiopathic small nerve fibre disease, diabetic neuropathy and healthy human subjects

**DOI:** 10.1111/j.1365-2559.2007.02851.x

**Published:** 2007-11

**Authors:** E P Wilder-Smith, W-Y Ong, Y Guo, A W-L Chow

**Affiliations:** Departments of Medicine, Yong Loo Lin School of Medicine, National University of Singapore Singapore; 1 Departments of Anatomy, Yong Loo Lin School of Medicine, National University of Singapore Singapore

**Keywords:** diabetic neuropathy, epidermis, idiopathic small nerve fibre disease, keratinocytes, neuropathic pain, transient receptor potential vanilloid 1

## Abstract

**Aims:**

The transient receptor potential vanilloid 1 (TRPV1) plays an important role in mediating pain and heat. In painful neuropathies, intraepidermal TRPV1 nerve fibre expression is low or absent, suggesting that pain generated is not directly related to sensory nerve fibres. Recent evidence suggests that keratinocytes may act as thermal receptors via TRPV1. The aim was to investigate epidermal TRPV1 expression in patients with neuropathic conditions associated with pain.

**Methods and results:**

In a prospective study of distal small nerve fibre neuropathy (DISN; *n* = 13) and diabetic neuropathy (DN; *n* = 12) intraepidermal nerve fibre density was assessed using the pan axonal marker PGP 9.5 and epidermal TPVR1 immunoreactivity compared with controls (*n* = 9). Intraepidermal nerve fibres failed to show TRPV1 immunoreactivity across all groups. There was moderate and strong TRPV1 reactivity of epidermal keratinocytes in 41.8% and 6% for DISN, 32.9% and 2.9% for DN and 25.4% and 5.1% for controls, respectively. Moderate keratinocyte TRPV1 expression was significantly increased in DISN compared with controls (*P* = 0.01).

**Conclusion:**

Our study suggests that in human painful neuropathies, epidermal TRPV1 expression is mainly in keratinocytes.

## Introduction

Capsaicin, the primary pungent compound in ‘hot’ chili peppers, produces pain and inflammation when placed on skin or mucous membranes. These responses are a consequence of capsaicin activating the transient receptor potential vanilloid 1 (TRPV1), a non-selective cation channel receptor within C and A δ nociceptors.^1^ Several reports have demonstrated the existence of TRPV1 in sensory neurons^2^,^3^ and the cloning of rat TRPV1 was a breakthrough in understanding its properties and physiological functions.^4^ TRPV1 has now been found to be widely distributed amongst different types of tissues and has been identified in the human brain,^5^ kidney,^6^ bronchial epithelial cells^7^ and recently in human keratinocytes in the epidermis.^8^ TRPV1, by its activation through noxious temperature, protons and cannabinoid chemicals, plays an increasingly recognized role in the biology of dermatological and neuropathic conditions,^8^ with anatomical distribution and disease influencing expression. TRPV1 expression in keratinocytes has recently led researchers to propose that keratinocytes may act as thermal receptors.^9^ TRPV1 has been shown to be essential for the modalities of pain sensation and thermal hyperalgia.^10^ However, its role in the pathogenesis of neuropathic pain, such as occurs in peripheral neuropathies, remains controversial. Although one study in a patient with postherpetic neuralgia has shown increased TRPV1+ intraepidermal nerve fibres (IENF), a recent study in patients with painful neuropathy found that TRPV1+ IENF were reduced and sometimes completely absent, suggesting that TRPV1 in epidermal axons is not primarily involved in the pathogenesis of neuropathic pain.^11^ Rather, it has been suggested that the skin as a whole may act as a polymodal nociceptor which undergoes functional changes in painful conditions.^11^ We set out to study the expression of epidermal TRPV1 in human skin by investigating two different types of neuropathy compared with healthy controls. Idiopathic small nerve fibre neuropathy was chosen because spontaneous heat pain is one of its primary characteristics, diabetic neuropathy (DN) because this is a common cause of sensory neuropathy.

## Materials and methods

In a prospective study, hypothenar intraepidermal nerve fibre density (IENFD) from healthy subjects and from subjects referred for investigation of neuropathy was assessed.^12^ In the subgroup reported here, the distribution of TPVR1 in the epidermis of the human skin is described. We studied the different epidermal TPVR1 staining intensity and distribution of patients with length-dependant DN and distal idiopathic small nerve fibre neuropathy (DSFN) and compared these with healthy subjects.

### subjects

After informed consent, those patients were included who had length-dependent DN with glove and stocking sensory symptoms (hypoaesthesia and/or dysaesthesia) with reduced touch perception to cotton wool and vibration sensation (128 Hz tuning fork) occurring in the presence of diabetes and who were without pathological levels of vitamin B12, thyroid-stimulating hormone and T4. Patients with sensory symptoms, but normal sensory testing to superficial touch and vibration were excluded.

Distal idiopathic small nerve fibre disease (DISN) was diagnosed in the presence of paraesthesiae (abnormal sensory perception) with additional findings of distal predominant small nerve fibre dysfunction (increased thermal and pin-prick sensation) on neurological examination.^13^ On sensory testing the paraesthesiae are typically painful. Sensory symptoms had to occur without neurophysiological and clinical evidence of large nerve fibre disease (normal ulnar and median nerve sensory motor nerve conduction, normal sural nerve conduction and normal peroneal motor nerve conduction and normal monofilament testing (200 mg) for all five digit tips bilaterally. Detailed history and tests excluded diabetes mellitus, amyloidosis, toxic substances (alcohol, lead) and inherited sensory and autonomic neuropathies.

Healthy subjects were recruited from hospital staff and students from the National University of Singapore. One neurologist (E.P.W.-S.) examined all healthy subjects to exclude neuropathy based on the absence of clinical features of sensory and motor symptoms and a normal sensory motor examination. Inclusion was furthermore dependent upon a negative history for diabetes, alcoholism, radiculopathies, ulnar or median nerve entrapments and no previous exposure to chemotherapy or other neurotoxic medications. Subjects <21 years old, with hand sepsis or ulceration were excluded.

## Procedures

All subjects gave informed consent and the National University Hospital Institutional Review Board approved the study. Hospital ethical guidelines are in accordance with the 1964 Declaration of Helsinki.

### skin biopsy specimens

Skin biopsy specimens were taken from the hypothenar region of the non-dominant hand using a 3-mm punch after local infiltration with 2% lignocaine, as previously described.^12^ Quantification of epidermal TRPV1 expression was performed using computerized image analysis (Image-Pro® Plus software; Media Cybernetics®, Silver Spring, MD, USA) linked to an upright microscope (Olympus® BX60, DP70 digital camera). The whole of the epidermis including basal and suprabasal areas was evaluated.

### immunocytochemistry

The skin biopsy specimens were cryoprotected by immersion in 15% sucrose and sectioned at 30 μm thickness using a cryostat. Sections were mounted on glass slides, washed in five to six 1-h changes of phosphate-buffered saline (PBS) and incubated for 1 h in a solution of serum (Vector, Burlingame, CA, USA) to block non-specific binding of the antibody. This was followed by incubation overnight in affinity-purified rabbit polyclonal antibodies to neuropeptide protein gene product (PGP) 9.5 or TRPV1. PGP 9.5 is found in vertebrate neurons and neuroendocrine cells and is commonly used to detect intraepidermal nerve fibres.^14^ The antibody to TRPV1 was purchased from Affinity Bioreagents (Golden, CO, USA). The sections were rinsed with PBS and incubated for 1 h at room temperature in a 1:200 dilution of biotinylated goat antirabbit IgG (Vector), followed by three changes of PBS to remove non-reacting secondary antibody. The sections were then reacted for 1 h at room temperature with an avidin-biotinylated horseradish peroxidase complex. The bound antibodies were visualized by treatment for 5 min in 0.05% 3,3,diaminobenzidine tetrahydrochloride and 0.2% nickel ammonium sulphate in Tris buffer with 0.05% hydrogen peroxide. The colour reaction was stopped with several washes of Tris buffer, followed by PBS. Sections were mounted on gelatin-coated glass slides, dehydrated and lightly counterstained with methyl green before cover-slipping. Control experiments replaced the primary antibody with PBS or non-immune rabbit IgG serum fraction. The experiments were performed on the skin of the hypothenar region and showed lack of immunoreactivity.

### intraepidermal nerve fibre density assessment

IENFD assessment was achieved by staining skin obtained from the hypothenar region with PGP 9.5, as previously described.^12^

### quantification of trpv1 receptor staining in keratinocytes

**Figure 1:**
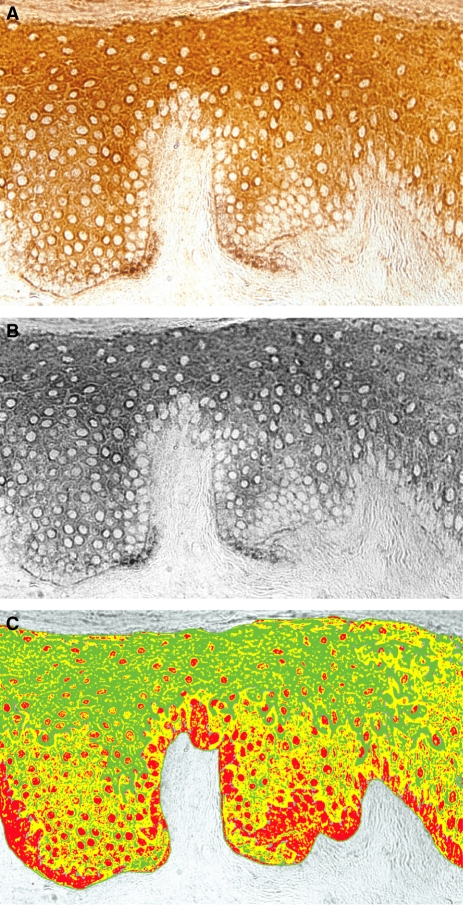
**A**, standard skin transient receptor potential vanilloid 1 (TRPV1) immunoreactivity in a patient with distal idiopathic small nerve fibre disease. **B**, the same image converted to black and white with eight levels of monochrome grey tones. **C**, the same image further converted into colour coding: green represents the most intense immunoreactivity, yellow moderate reactivity and red immunonegativity. (TRPV1 peroxidase stain.)

Assessment of immunoreactivity with TRPV1 was performed using a previously described and standardized semiquantitative assessment of photographs taken from antibody-labelled sections.^15^ Using Image-Pro® Plus software, the colour image was converted into a black-and-white image with eight levels of monochrome grey tones (Figure 1). This picture was termed the ‘filter’. The operator then selected the cut-off level of the perceived limit of TRPV1 staining. The operator was aided in this selection by a diagrammatic scale of the range of staining intensities seen in the ‘filter’. The software program arbitrarily attaches a number ranging from 0 to 225 across all intensities of immunoreactivity, with absent (visually perceived as white) reactivity being ranked 225. The chosen numerical values used to assign intensity of reactivity with the Image-Pro® Plus software were 155–225 for absent staining (equivalent to immunonegativity); 154–124 for moderate and 123–0 for the highest level of immunoreactivity. Moderate and intense reactivity was selected by the operator at levels thought to represent moderate and intense immunoreactivity of cells. The 155–225 range (equivalent to immunonegativity) was assigned a red colour, 154–124 (moderate reactivity) yellow and 123–0 (highest immunoreactivity) green (Figure 1). The Image-Pro® Plus software was then used to calculate the areas of the different intensities of TRPV1 reativity of the epidermal tissue. The data were formulated as the percentage of epidermal tissue without and with moderate and strong immunoreactivity for TRPV1. Measurements were performed for five fields per patient chosen at random from two sections oriented longitudinally (425 μm). The operator delineated the field of epidermal skin to be assessed by the software (Figure 1). The percentage area of TRPV1 keratinocyte immunoreactivity was then automatically derived for the whole epidermal region as well as separately for the suprabasal and basal compartments. This subdivision was designed to assess separately the more actively dividing basal cell layers from the more differentiated keratinocyte cells located higher in the epidermis.^16^ The suprabasal epidermal compartment was taken as the top 2/3 region in the absence of anchoring papillae extending into the dermis. For anchoring papillae two cell layers superior to the basal laminae were included. The mean suprabasal and basal epidermal regions of absent, moderate and intense TRPV1 reactivity were obtained for each subject and subsequently summarized as means for each of the three groups.

The following photographic conditions applied: sensitivity ISO200, exposure time 1/18 s, objective enlargement ×40, exposure mode manual and spot 30%, photo size 4080 × 3072 μm.

### analysis

One-way anova was used to calculate differences in intensity of reactivity between the three groups for the epidermis as a whole and subdivided into suprabasal and basal compartments. *P* < 0.05 was considered to be significant.

## Results

We examined 13 patients (nine female; mean age 49 ± 13.5 years, range 22–65) with DISN, 12 patients (four female; mean age 51 ± 17.3 years, range 24–73) with DN and nine healthy subjects (six female; mean age 45 ± 11.9 years, range 22–61).

### clinical data

The mean blood sugar level of the diabetic patients was 9.5% (range 5.9–13.8%). In our laboratory, non-diabetic levels are <6.4%, optimal diabetic control is 6.5–7.0% and poor diabetic control is >8.0%. Six of the patients with idiopathic small nerve fibre disease and three of those with diabetes complained of a spontaneous or intermittent burning sensation in the hands or feet at the time of the biopsy. It was not possible to estimate accurately the length of time the burning sensation had been present.

### pgp 9.5 staining for axons

Normal numbers of epidermal nerves were observed in control patients, and significantly (*P* < 0.05) fewer nerve fibres were seen in idiopathic small nerve fibre disease and DN (Table 1).

**Table 1:** Mean percentage and degree of TRPV1 expression for cells within the epidermis

Groups	*n*	IENFD	Mean % absent TRPV1 expression	Mean % moderate TRPV1 expression	Mean % strong TRPV1 expression
DISN	13	1.5 (±1.8)	52.2 (±19.9)	41.8 (14.3)*	6 (7.5)
DN	12	0.8 (±1.1)	64.2 (±16.6)	32.9 (13)	2.9 (4.3)
Controls	9	2.6 (±2.2)	69.5 (±20.6)	25.4 (17.4)	5.1 (5.7)

DISN, distal small nerve fiber disease; DN, diabetic neuropathy; IENFD, intraepidermal nerve fibre density.

The numbers in bracket represent the standard deviation.

* Indicates a significant difference of *P* = 0.015 between small nerve fibre and healthy groups.

### trpv1 immunohistochemistry

**Figure 2:**
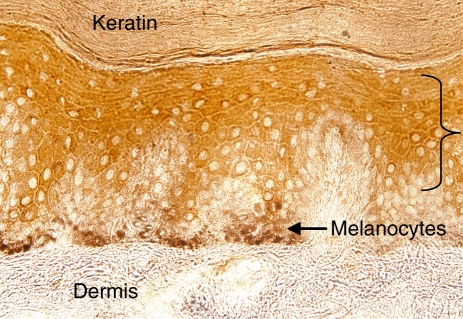
Maximal transient receptor potential vanilloid 1 (TRPV1) immunoreactivity is seen in the keratinocytes of the suprabasal layer of the epidermis. The parentheses indicate the suprabasal region. Note the lack of nuclear TRPV1 staining. (TRPV1 peroxidase stain.)

The epidermis showed heterogeneous immunoreactivity, with increased reactivity occurring more in keratinocytes of the suprabasal layer of the epidermis (Figure 2). Tables 1–3 show the intensity of reactivity of the three different clinical groups according to analysis of the whole epidermal region and subdivision of the epidermis into suprabasal and basal regions. A statistically significant increase in TRPV1 immunoreactivity was observed in keratinocytes of patients with idiopathic small nerve fibre disease. TRPV1 expression was significantly increased in the whole epidermal region in DISN when compared with controls (*P* = 0.01). Further analysis showed that the suprabasal areas were consistently more immunoreactive than basal regions. Keratinocyte reactivity (moderate) for TRPV1 in the suprabasal region was significantly increased (*P* = 0.005) in DISN compared with healthy groups, but failed to reach significance for the diabetic group and when extending analysis to the basal region. Keratinocyte reactivity was mostly cytoplasmic, with little or no reactivity of the nucleus (Figure 2).

**Table 2:** Mean percentage of TRPV1 expression for cells within the suprabasal epidermal regions

Groups	*n*	Mean % absent TRPV1 expression	Mean % moderate TRPV1 expression	Mean % strong TRPV1 expression
DISN	13	44.6 (±22.4)	48.1 (15.9)*	7.3 (9.2)
DN	12	66.7 (±19.1)	39.5 (14.3)	3.8 (6)
Controls	9	66.9 (±22.7)	27.3 (17.9)	5.8 (6.4)

* A significant difference of *P* = 0.005 between small nerve fibre and healthy groups.

DISN, distal small nerve fibre disease; DN, diabetic neuropathy.

**Table 3:** Mean percentage of TRPV1 expression for cells within the basal epidermal regions

Groups	*n*	Mean % absent TRPV1 expression	Mean % moderate TRPV1 expression	Mean % heavy TRPV1 expression
DISN	13	73.4 (±13.9)	24.3 (11.6)	2.3 (2.8)
DN	12	82.3 (±14.2)	16.9 (13.1)	0.8 (1.2)
Controls	9	80.2 (±17.2)	17.3 (14.9)	2.5 (3.3)

DISN, distal small nerve fibre disease; DN, diabetic neuropathy.

## Discussion

This study reports on the epidermal distribution of TRPV1 in the glabrous hand skin from healthy controls, patients with DISN and DN. The most important finding was the increased keratinocyte immunoreactivity to TRPV1 in patients with DISN in the absence of IENF immunoreactivity with TRPV1. For many years it was thought that TRPV1 was expressed primarily in peripheral sensory neurons.^17^ Studies performed both in the skin of humans and rodents have carefully investigated the distribution of TRPV receptors in nerve tissue and across different tissues.^8^,^18^ Stander’s study investigating the distribution of TRPV1 in cutaneous sensory nerves of human skin pointed out that in healthy humans, IENF do not or only poorly immunoreact with TRPV1.^8^ Skin areas with strong TRPV1 reactivity were the dermal sensory axons, hair follicles, blood vessels and keratinocytes. In contrast, a recent study has found TRPV1 expression throughout the peripheral nervous system, including intraepidermal fibres.^11^ The IENF immunonegativity in both our study and that of Stander *et al.* may be due to differences in antibody specificity. Although lower specificity of the TRPV1 antibodies used in our study and that of Stander *et al.* may have played a role, this seems a moot point considering Lauria’s recent study showing reduced or absent TRPV1 IENF density in the epidermis of patients with neuropathic pain.^11^

The finding of this study that keratinocyte TRPV1 is significantly increased in patients with DISN suggests that one of the primary characteristics of DISN–spontaneous heat pain – may be related to increased keratinocyte TRPV1. The robust evidence of strong TRPV1 expression in keratinocytes^9^ has led to the belief that keratinocytes are capable of functioning as one of the main thermosensory receptors.^19^ Studies using cultured human epidermal keratinocytes have confirmed that expressed TRPV1 responds to heat and noxious stimuli^20^ and that activation of epidermal keratinocyte TRPV1 results directly in release of cyclooxygenase-2, one of the main mediators of inflammation.^21^

The finding of reduced IENFD in our patients with DISN further supports the notion that the IENF themselves do not play a primary role in the production of heat sensation. To our knowledge, only one study has demonstrated increased TRPV1 immunoreactivity in a patient with painful neuropathy (postherpetic neuralgia).^22^ It is interesting to note that although the authors report a somewhat patchy increase of IENF with increased TRPV1, the immunofluorescence also seems clearly to show increased keratinocyte expression in the skin afflicted with neuropathy. The authors propose that pain is maintained by a peripheral nociceptive maladaptation located within the skin, a theory apparently supported by neuropathic pain directly and drastically responding to excision of the painful skin.

The association of increased keratinocyte TRPV1 in human conditions with pain has recently been demonstrated in women with breast pain, in patients with rectal hypersensitivity and faecal urgency, as well as in pruritic skin of patients with prurigo nodularis.^8^,^23^,^24^ We recently described a patient with steroid-responsive small nerve fibre symptoms of burning feet and hands, where keratinocytes showed strong TRPV1 expression.^25^ On follow-up skin biopsy after steroid administration, keratinocyte TRPV1 expression was drastically reduced in parallel with remission of clinical symptoms (manuscript in preparation).

The semiquantitative technique employed in this study has shown that in the epidermis of the healthy human glabrous skin about one-third of the keratinocytes show moderate TRPV1 expression, around 5% intensely so. The suprabasal epidermal area showed highest expression across all three groups, suggesting that expression may be linked to greater differentiation of keratinocytes, which is least in the basal epidermal region.^16^ This contrasts with the study by Stander *et al.*, which found the highest expression in the basal keratinocytes.^8^ This may be due to differences in TRPV1 antibody specificity, in addition to the different skin type (glabrous) examined in the present study.

Our study has several shortcomings. Because of small numbers, it was not possible to perform statistical analysis to analyse the effect of burning compared with no burning across the groups or within groups. This would be of considerable interest in future studies. Furthermore, neuropathic pain or thermal hypersensitivity was also seen in some of the patients with DN. This overlap of clinical symptoms may help to explain why the mean keratinocyte TRPV1 expression in the DN group was also higher compared with controls.

In conclusion, our data show that increased keratinocyte TRPV1 expression in patients with small nerve fibre neuropathy and, to a lesser extent, in DN may play a role in explaining some of the typical clinical features of increased sensitivity to noxious stimuli.

## Abbreviations:

DISN: distal idiopathic small nerve fibre disease
DN: diabetic neuropathy
DSFN: distal idiopathic small nerve fibre neuropathy
IENF: intraepidermal nerve fibres
IENFD: intraepidermal nerve fibre density
PBS: phosphate-buffered saline
PGP: protein gene product
TRPV1: transient receptor potential vanilloid 1
